# Bicycle spiroergometry: comparison of standardized examination protocols for adolescents: is it necessary to define own standard values for each protocol?

**DOI:** 10.1007/s00421-021-04601-y

**Published:** 2021-03-12

**Authors:** Jana Windhaber, Monica Steinbauer, Magdalena Holter, Annemarie Wieland, Kristina Kogler, Regina Riedl, Peter Schober, Christoph Castellani, Georg Singer, Holger Till

**Affiliations:** 1 Department of Paediatric and Adolescent Surgery, Medical University of Graz, Auenbruggerplatz 34, 8036 Graz, Austria; 2 Institute for Medical Informatics, Statistics and Documentation, Medical University of Graz, Graz, Austria

**Keywords:** Spiroergometry, Adolescents, Examination protocols, VO_2_peak, Maximal power, O_2_ pulse, OUES, Ventilatory threshold, Lactate threshold

## Abstract

**Purpose:**

To compare performance data of adolescents collected with five different bicycle spiroergometry protocols and to assess the necessity for establishing standard values for each protocol.

**Methods:**

One-hundred-twenty adolescents completed two bicycle spiroergometries within 14 days. One of the two tests was performed based on our institutional weight-adapted protocol (P0). The other test was performed based on one out of four exercise protocols widely used for children and adolescents (P1, 2, 3 or 4) with 30 persons each. The two tests were performed in a random order. Routine parameters of cardiopulmonary exercise tests (CPET) such as VO_2_peak, maximum power, O_2_ pulse, OUES, VE/VCO_2_ slope as well as ventilatory and lactate thresholds were investigated. Agreement between protocols was evaluated by Bland–Altman analysis, coefficients of variation (CV) and intra-class correlation coefficients (ICC).

**Results:**

None of the CPET parameters were significantly different between P0 and P1, 2, 3 or 4. For most of the parameters, low biases between P0 and P1–P4 were found and 95% confidence intervalls were narrow. CV and ICC values largely corresponded to well-defined analytical goals (CV < 10% and ICC > 0.9). Only maximal power (Pmax) showed differences in size and drift of the bias depending on the length of the step duration of the protocols.

**Conclusion:**

Comparability between examination protocols has been shown for CPET parameters independent on step duration. Protocol-dependent standard values do not appear to be necessary. Only Pmax is dependent on the step duration, but in most cases, this has no significant influence on the fitness assessment.

## Introduction

Cardiopulmonary exercise testing (CPET) is the gold standard for determining aerobic fitness in medicine. It provides information describing the function of respiratory, cardiocirculatory, neuromuscular, blood, and metabolic systems, as well as limits of exercise tolerance and thus is useful in the diagnosis, management, and prognosis of diseases and sports medicine issues (Cooper et al. 2014). Since the physiological responses to exercise change during growth and development, appropriate pediatric reference values seem essential for adequate interpretation of CPET.

Two recent reviews have described significant heterogeneity between examination protocols (e.g., step duration and increment) and suggested adjustment for body size or weight (Blais et al. 2015; Pianosi et al. 2017). The authors pointed out that the quality criterion of comparability is not met due to the large number of different applied protocols. Therefore, recommendations conclude that each protocol needs its own set of reference values (Paridon et al. 2006).

VO_2_peak in adolescents and adults seems to be a robust variable that is normally independent of the exercise protocol (Armstrong and McManus 2017; Sheehan et al. 1987; Welsman et al. 2005). Nevertheless, there is lacking information about the comparability of parameters in the submaximal range. These values become increasingly important in pediatric performance diagnostics since achievable maximum values depend on the motivation of the test subject.

Testing duration represents the variable with the largest consensus in the literature. A plethora of different studies has indicated an optimum duration for a maximal cardiopulmonary exercise test of 8–12 min for adolescents and adults (Buchfuhrer et al. 1983; Hebestreit 2004; Myers et al. 1989; Takken et al. 2017; Whipp et al. 1981; Yoon et al. 2007).

The objective of this study was therefore to examine performance data collected with different bicycle spiroergometry protocols and to assess the necessity for establishing standard values for each protocol. Furthermore, the test efficiency of each examination protocol was evaluated in terms of required duration of the spiroergometry.

## Methods

One-hundred-twenty adolescents (14–18 years) of both genders (60 males, 60 females) completed two bicycle spiroergometries with measurement of lactate until subjective exhaustion. The median interval between the two examinations was 9 days and ranged between 2 and 14 days. One of the two tests was performed considering a weight-adapted 1-min protocol of Windhaber and Schober (P0) developed and applied in the testing entity of sports medicine at the Department of Paediatric and Adolescent Surgery, Medical University of Graz. The other test was performed applying one of the exercise protocols widely used for children and adolescents [Godfrey–Protocol (P1) cited in Hebestreit (Hebestreit et al. 2002)], stress protocols recommended by the sports association (P2 and P3) or the protocol of Rost and Hollmann (P4) cited in Hebestreit (Hebestreit et al. 2002). Details of the different protocols are summarized in Table 1.

**Table 1:** Investigation protocols

Group 1–*N* = 30 P 1 (Godfrey-Protocol)	Initial step Watt	Increment Watt	Duration Minutes	Recovery Watt
Persons up to 150 cm	15	15	1	15
Persons greater than 150 cm	20	20	1	20

Group 2–*N* = 30 P 2 (Austrian Ski Federation)	Initial step Watt	Increment Watt	Duration Minutes	Recovery Watt
Male	70	30	2	70
Female	50	25	2	50

Group 3–*N* = 30 P 3 (Austrian Cycling Federation)	Initial step Watt	Increment Watt	Duration Minutes	Recovery Watt
Male	50	50	3	50
Female	30	30	3	30

Group 4–*N* = 30 P 4 (Protocol of Rost/Hollmann)	Initial step Watt/kg	Increment Watt/kg	Duration Minutes	Recovery Watt/kg
Male	0.5	0.5	2	0.5
Female	0.5	0.5	2	0.5

All Athletes (Group 1–4)–*N* = 120 P 0 (Protocol of Windhaber/Schober)	Initial step Watt	Increment Watt	Duration Minutes	Recovery Watt
Male and female				
25–30 kg	15	10	1	15
31–35 kg	20	10	1	20
36–40 kg	25	10	1	25
41–45 kg	30	15	1	30
46–50 kg	35	15	1	35
Female				
51–55 kg	40	15	1	40
56–60 kg	45	15	1	45
> 60 kg	50	20	1	50
Male				
51–55 kg	40	20	1	40
56–60 kg	45	20	1	45
61–70 kg	50	25	1	50
> 70 kg	50	30	1	50

The participants were divided into 4 groups of 30 subjects each (15 males, 15 females). Group 1 was investigated with protocols P0 and P1, group 2 with protocols P0 and P2, group 3 with protocols P0 and P3 and group 4 with protocols P0 and P4. The order in which the two tests were performed was randomly assigned.

Conditions of participation were no infectious disease at least 14 days before the respective examination date, no chronic illness, ban on sports the day before and on the examination day and a light meal 2–3 h before the test.

CPET was performed in the upright position with a cycle ergometer (Excalibur Sport®, Lode B.V., Groningen, The Netherlands). Minute ventilation (VE), O_2_ uptake (VO_2_) and CO_2_ production (VCO_2_) were measured with a calibrated respiratory gas analysis system (Oxycon Pro®, Carl Reiner GmbH, Vienna, Austria). Heartrate was measured by continuous twelve-lead electrocardiography (Cardinal Health™ electrocardiography, Dublin, Ireland). Lactate levels were obtained collecting 20 μl blood of the earlobe prior to the test and at the end of each step (Biosen C_line®, EKF Diagnostics for life, Cardiff, UK).

All tests were supervised by a physician of sports medicine and a biomedical scientist. The participants were verbally encouraged to continue the investigation until exhaustion, as the participants were unable to maintain the required cadence of more than 60 revolutions per minute (rpm). A respiratory exchange rate (RER) > 1.10 was used as criterion to determine that VO_2_peak represents a physiological peak workload (Armstrong and van Mechelen 2008; Mezzani et al. 2009, 2003). All subjects included in the study had a RER above 1.10.

Informed written consent was obtained from all athletes and their legal guardians. The investigation conforms to the Code of Ethics of the World Medical Association (Declaration of Helsinki). The study was approved by the institutional review board (EK 30–187 ex 17/18). Patients or the public were not involved in the design, or conduct, or reporting, or dissemination plans of our research.

### Measured and calculated parameters

We have selected parameters that should be part of a routine CPET as per the latest recommendations (Paridon et al. 2006). Additionally, submaximal parameters and slopes were added as previously published (Dallaire et al. 2017).

#### Maximal values

Maximal Heart Rate (HRmax) was defined as the highest heart rate achieved during exercise and expressed in bpm. Maximal Power (Pmax) is presented as Watt per bodyweight (W/kg). In case of incomplete step duration, the maximal work rate was calculated by linear extrapolation based on time. Peak oxygen uptake (VO_2_peak) was defined as the average value over the last 30 s prior to termination of the test and is expressed in ml/kg/min. O_2_ pulse was measured as VO_2_/HR and expressed in ml/beat.

#### Ventilatory and lactate thresholds

Ventilatory threshold (VT) was estimated according to the V-slope method (Beaver et al. 1986). The individual anaerobic lactate threshold (IAT) was defined as the VO_2_ value at the intersection point of the tangent to the lactate curve at the point of lactate maximum with the abscissa (time axis), as previously described in the literature (Pessenhofer et al. 1990; Schwaberger et al. 1985). Two- and 4-mmol thresholds were calculated as described by Mader (Mader and Heck 1986). All thresholds were expressed as values of VO_2_ in ml/kg/min.

#### Slopes

From gas exchange measurements (VE, VO_2_, VCO_2_), slopes were calculated using standard linear regression as described in the literature (Cooper et al. 2014; Sun et al. 2012). VE versus VCO_2_ (dVE/dVCO_2_) was calculated as the slope obtained by linear regression analysis of VE (l/min) versus VCO_2_ (ml/min).

Oxygen uptake efficiency slope (OUES) was calculated by a linear least square regression of the VO_2_ (ml/min) versus the common logarithm of VE (l/min) under consideration of all available measurement points (Baba et al. 1996).

#### Duration of test time

The duration of test time is expressed in minutes. For each protocol, the percentage of performed tests in the target range of 8–12 min is given.

### Statistics

Descriptive statistics are presented as absolute and relative frequencies for categorical data and as means and standard deviations (SD) or medians and ranges for continuous data. For performance data, agreement between different protocols was evaluated by Bland–Altman analysis, coefficients of variation (CV) and intra-class correlation coefficients (ICC) for every group separately. For the Bland–Altman analysis, 95% confidence intervals (CI) for the bias (difference in parameters between P0 and the corresponding protocol) as well as the upper and lower limits of agreement are presented. The CV was calculated as SD of the bias (P0 minus other protocols, respectively) divided by the mean of the protocols and is presented as a percentage, i.e., CV * 100. This calculation of the CV is commonly used in sport medicine e.g., in Tompuri et al. 2016. The ICC and its 95% CI were assessed by a two-way random model (ICC [2,1] concept). Differences in performance data between the protocols were analyzed by pairwise Wilcoxon rank-rum tests due to non-normally distributed differences. Test durations of the protocols were compared by a paired t-test. The statistical analysis was performed with SAS software (version 9.4; SAS Institute, Inc). Statistical significance was set to *α* = 5%.

## Results

### Anthropometric characteristics

The mean age of the 120 participants was 15.7 years (range: 14–18 years). The anthropometric characteristics are presented in Table 2. All values were within national references. There were no differences between the groups.

**Table 2:** Anthropometric characteristics

Groups	Variable	Mean	Standard deviation	Median	Minimum	Maximum
Group 1 *N* = 30	Weight (kg)	60.27	10.88	59.50	42.00	88.00
	Height (m)	1.72	0.10	1.72	1.56	1.91
	BMI (kg/m^2^)	20.26	2.19	19.94	16.33	24.12
Group 2 *N* = 30	Weight (kg)	67.87	11.90	69.00	50.00	97.00
	Height (m)	1.71	0.10	1.69	1.55	1.87
	BMI (kg/m^2^)	23.03	2.61	22.73	18.82	29.61
Group 3 *N* = 30	Weight (kg)	60.83	9.64	60.00	42.00	77.00
	Height (m)	1.71	0.10	1.71	1.54	1.88
	BMI (kg/m^2^)	20.69	1.80	20.35	17.26	24.46
Group 4 *N* = 30	Weight (kg)	61.00	11.41	59.50	34.00	84.00
	Height (m)	1.70	0.10	1.72	1.50	1.92
	BMI (kg/m^2^)	20.88	2.56	20.14	15.11	28.07

### Maximal values, thresholds and slopes

The mean, standard deviation, median, and range of parameters measured with P0 and with the protocol selected for the corresponding group 1–4 are shown in Table 3 for comparison. There were no statistically significant differences of the measurement results between P0 and P1, 2, 3 or 4. Details on biases, 95% CIs, bounds, CV- and ICC-values are presented in Table 4.

**Table 3:** Mean, standard deviation, median and range of maximal values, thresholds and slopes (Group 1 was investigated with protocols P0 and P1, group 2 with protocols P0 and P2, group 3 with protocols P0 and P3 and group 4 with protocols P0 and P4. N = 30 per group)

			Group	Protocol 0				Protocol 1, 2, 3 or 4			
				Mean	SD	Median	Range	Mean	SD	Median	Range
HR max min^−1^			1	194.4	6.9	194.0	27.0	192.8	7.4	191.0	31.0
			2	198.1	7.4	197.0	27.0	198.2	8.5	198.5	33.0
			3	194.2	10.8	194.0	45.0	195.3	12.4	198.5	55.0
			4	195.5	9.2	195.0	34.0	194.6	8.2	195.0	37.0
Pmax/kg Watt/kg			1	4.0	0.6	4.1	2.4	4.0	0.6	4.1	2.4
			2	4.1	0.7	4.2	3.1	3.7	0.6	3.7	2.7
			3	4.4	1.1	4.4	4.2	3.9	1.0	3.9	3.4
			4	3.8	0.5	3.7	1.8	3.5	0.4	3.5	1.7
VO_2_peak/kg ml/kg/min			1	49.9	6.5	49.6	23.4	49.7	7.5	51.2	29.1
			2	49.3	7.1	51.0	30.8	50.2	8.0	51.7	33.8
			3	51.4	10.0	52.6	35.5	51.2	10.5	54.0	35.8
			4	48.3	4.7	47.9	18.6	48.4	5.8	47.8	24.1
O_2_ Pulse ml/min^−1^			1	15.5	3.5	15.5	12.9	15.6	3.9	15.9	14.6
			2	17.0	4.1	16.5	17.6	17.3	4.3	17.0	19.2
			3	16.4	4.7	15.5	14.5	16.2	4.8	15.7	15.8
			4	15.0	3.0	14.7	11.6	15.2	3.2	15.0	11.0
VO_2_/kg_VT ml/kg/min			1	20.1	4.0	19.7	18.4	20.3	3.2	20.2	15.3
			2	18.2	3.0	17.8	12.6	18.1	2.5	17.8	11.9
			3	24.8	7.8	25.0	24.9	24.5	7.4	24.0	25.3
			4	20.0	3.2	20.1	12.8	19.7	3.2	19.3	13.2
VO_2_/kg_2mmol ml/kg/min			1	27.8	4.8	28.5	21.0	28.2	4.5	28.2	16.8
			2	27.9	5.2	27.3	23.5	29.7	5.9	30.7	26.1
			3	33.9	9.3	31.7	33.0	33.4	8.9	33.3	32.0
			4	28.3	4.8	27.3	20.8	28.4	5.0	28.4	17.6
VO_2_/kg_4mmol ml/kg/min			1	38.8	8.7	38.8	46.8	37.6	5.4	37.7	18.4
			2	36.8	5.2	36.9	26.2	38.0	6.3	38.5	29.9
			3	42.1	9.7	42.0	32.2	41.8	9.4	43.3	33.9
			4	37.7	4.9	36.5	20.8	37.3	5.2	37.1	18.9
VO_2_/kg_IAT ml/kg/min			1	35.1	5.4	34.7	23.5	35.5	6.0	36.4	23.3
			2	36.7	6.3	38.5	27.9	38.5	7.1	39.4	31.0
			3	40.5	10.9	41.4	39.1	39.3	10.6	40.3	37.5
			4	34.1	4.8	33.8	19.2	34.2	6.4	33.2	24.9
Slope VE/VCO_2_			1	27.7	3.4	27.3	14.9	28.8	4.3	28.9	22.2
			2	29.1	4.2	28.4	18.1	27.3	4.0	27.2	20.7
			3	30.3	5.2	30.3	28.3	29.9	5.0	28.8	24.7
			4	30.7	4.2	30.6	16.5	31.1	3.9	31.3	14.0
OUES			1	1256.4	277.7	1249.9	1028.2	1275.8	293.1	1283.0	1118.3
			2	1426.7	343.6	1451.2	1430.9	1473.7	359.0	1472.5	1370.5
			3	1388.1	397.9	1311.0	1401.8	1401.4	419.6	1360.3	1539.0
			4	1283.1	207.8	1278.6	825.4	1299.7	255.4	1290.5	946.7

**Table 4:** Comparison P0 with P1-4: Bias, 95% CI, lower and upper bound, differences in %, CV, ICC and 95% CI of maximal values, thresholds, and slopes

Variable	Comparison P0 with Protocol		Bias	95% CI	Lower bound	Upper bound	Diff in %	Lower bound %	Upper bound %	CV%	ICC	95% CI
HR max min^−1^		P1	1.6	(0, 3.1)	− 6.7	9.9	0.8	− 3.4	5.1	2.2	0.81	(0.64, 0.90)
		P2	− 0.1	(− 1.5, 1.4)	− 7.7	7.6	0	− 3.9	3.8	2.0	0.88	(0.77, 0.94)
		P3	− 1.1	(− 3.4, 1.2)	− 13.4	11.2	− 0.5	− 6.9	5.9	3.2	0.86	(0.73, 0.93)
		P4	0.9	(− 0.6, 2.4)	− 6.9	8.8	0.5	− 3.5	4.4	2.1	0.89	(0.79, 0.95)
Pmax/kg Watt/kg		P1	0.0	(− 0.1, 0.1)	− 0.5	0.5	0.3	− 13.0	13.5	6.5	0.90	(0.80, 0.95)
		P2	0.4	(0.3, 0.5)	− 0.1	0.8	9.4	− 0.8	19.7	5.7	0.80	(0.63, 0.90)
		P3	0.5	(0.4, 0.6)	− 0.0	1.0	11.5	− 0.6	23.5	6.4	0.88	(0.77, 0.94)
		P4	0.3	(0.2, 0.4)	− 0.1	0.7	7.7	− 3.5	19.0	5.8	0.74	(0.53, 0.86)
VO_2_peak/kg ml/kg/min		P1	0.2	(− 1.0, 1.4)	− 6.1	6.5	0.7	− 11.7	13.1	6.5	0.90	(0.80, 0.95)
		P2	− 0.9	(− 1.8, − 0.1)	− 5.5	3.6	− 1.6	− 10.6	7.4	4.7	0.95	(0.90, 0.98)
		P3	0.3	(− 0.9, 1.4)	− 5.9	6.4	0.8	− 12.2	13.8	6.2	0.95	(0.90, 0.98)
		P4	− 0.1	(− 1.3, 1.1)	− 6.2	6.1	0.1	− 12.5	12.6	6.5	0.83	(0.68, 0.91)
O_2_ Pulse ml/min^−1^		P1	− 0.1	(− 0.4, 0.3)	− 1.9	1.7	0	− 11.2	11.1	5.8	0.97	(0.94, 0.99)
		P2	− 0.4	(− 0.7, − 0.1)	− 2.0	1.3	− 2.1	− 11.1	7.0	4.9	0.98	(0.96, 0.99)
		P3	0.2	(− 0.1, 0.5)	− 1.4	1.8	1.5	− 7.9	10.8	4.9	0.99	(0.98, 1.00)
		P4	− 0.2	(− 0.6, 0.1)	− 2.0	1.6	− 1.2	− 12.7	10.3	6.1	0.95	(0.90, 0.98)
VO_2_/kg_VT ml/kg/min		P1	− 0.2	(− 0.9, 0.5)	− 3.8	3.4	− 1.7	− 19.7	16.4	9.1	0.88	(0.77, 0.94)
		P2	0.2	(− 0.3, 0.7)	− 2.6	3.0	0.6	− 15.0	16.2	7.8	0.87	(0.75, 0.93)
		P3	0.2	(− 0.5, 1.0)	− 3.5	4.0	0.7	− 16.6	18.0	7.8	0.97	(0.94, 0.99)
		P4	0.3	(− 0.3, 0.9)	− 3.0	3.6	1.6	− 15.8	18.9	8.4	0.87	(0.75, 0.93)
VO_2_/kg_2mmol ml/kg/min		P1	− 0.4	(− 1.8, 1.0)	− 7.8	7.0	− 1.5	− 26.0	23.1	13.5	0.68	(0.44, 0.83)
		P2	− 1.8	(− 2.8, − 0.8)	− 7.1	3.5	− 5.8	− 25.4	13.8	9.4	0.84	(0.70, 0.92)
		P3	0.4	(− 0.7, 1.5)	− 5.5	6.3	0.9	− 18.3	20.2	9.0	0.95	(0.90, 0.98)
		P4	− 0.2	(− 1.3, 1.0)	− 6.3	6.0	− 0.4	− 21.7	20.9	11.0	0.80	(0.63, 0.90)
VO_2_/kg_4mmol ml/kg/min		P1	1.2	(− 1.2, 3.6)	− 11.6	13.9	2.0	− 22.6	26.7	17.0	0.60	(0.32, 0.78)
		P2	− 1.2	(− 2.1, − 0.3)	− 5.9	3.4	− 2.7	− 16.0	10.6	6.3	0.90	(0.80, 0.95)
		P3	0.3	(− 0.6, 1.3)	− 4.8	5.5	0.7	− 12.0	13.4	6.3	0.96	(0.92, 0.98)
		P4	0.4	(− 0.6, 1.5)	− 5.1	6.0	1.3	− 13.1	15.6	7.6	0.85	(0.71, 0.92)
VO_2_/kg_IAT ml/kg/min		P1	− 0.5	(− 1.6, 0.7)	− 6.7	5.8	− 1.0	− 19.4	17.3	9.0	0.85	(0.71, 0.92)
		P2	− 1.8	(− 2.7, − 0.8)	− 6.7	3.2	− 4.2	− 18.1	9.7	6.8	0.90	(0.80, 0.95)
		P3	1.2	(0.3, 2.0)	− 3.5	5.8	2.9	− 10.0	15.8	6.0	0.97	(0.94, 0.99)
		P4	− 0.1	(− 1.3, 1.2)	− 6.7	6.5	0.6	− 19.4	20.5	9.8	0.83	(0.68, 0.91)
Slope VE/VCO_2_		P1	− 1.1	(− 2.2, − 0.1)	− 6.7	4.5	− 3.5	− 22.7	15.6	10.1	0.71	(0.48, 0.85)
		P2	1.8	(0.3, 3.3)	− 6.0	9.7	6.7	− 22.6	36.0	14.2	0.46	(0.14, 0.70)
		P3	0.4	(− 0.7, 1.5)	− 5.3	6.1	1.4	− 18.8	21.6	9.7	0.84	(0.70, 0.92)
		P4	− 0.3	(− 1.3, 0.6)	− 5.3	4.6	− 1.2	− 17.5	15.2	8.2	0.81	(0.64, 0.90)
OUES		P1	− 19.5	(− 61.0, 22.1)	− 237.5	198.5	− 1.3	− 17.5	14.9	8.8	0.92	(0.84, 0.96)
		P2	− 47.0	(− 99.5, 5.5)	− 322.4	228.4	− 3.0	− 26.6	20.6	9.7	0.91	(0.82, 0.96)
		P3	− 13.3	(− 59.1, 32.6)	− 254.1	227.5	− 0.6	− 17.3	16.1	8.8	0.96	(0.92, 0.98)
		P4	− 16.6	(− 50.1, 17.0)	− 192.7	159.6	− 0.7	− 14.9	13.5	7.0	0.93	(0.86, 0.97)

#### Differences in maximal values

The biases of HRmax between P0 and P1–P4 were approximately 1 beat/min with a 95% CI of − 3 to 3 beats/min. CV values were between 2 and 3% and ICC values were between 0.81 and 0.89.

The biases of VO_2_ peak were < 1 ml/kg/min, with a 95% CI from − 1.8 to 1.4 ml/kg/min. CV values were approximately 6% and ICC values were between 0.83 and 0.95.

The biases of O_2_ pulse were < 0.5 ml/beat, with a 95% CI from − 0.7 to 0.5 ml/beat. CV values were between 5 and 6% and ICC values ranged from 0.95 to 0.99.

Although the differences in Pmax were also not statistically significant, there were differences in the size and drift of the bias depending on the length of the step duration of the protocols. In group 1, when comparing the protocols P0 and P1, both with a step duration of 1 min but different increments, the bias was 0.0 with a 95% CI of − 0.1 to 0.1 W/kg. In group 2, the average power measured with P0 was 0.4 W/kg higher (95% CI 0.3–0.5) than the power measured with P2 with step duration of 2 min, which corresponds to 9.4% of maximum power. In group 3, comparing P0 with P3 with a step duration of 3 min, the average power was 0.5 W/kg higher with P0 (95% CI 0.4–0.6), which corresponds to 11.5% of maximum power. In group 4, the average power measured with P0 was 0.3 W/kg higher (95% CI 0.2–0.4) than the power measured with P4 with step duration of 2 min, which corresponds to 7.7% of maximum power. CV values of all groups were similar at approximately 6%, with an ICC of 0.74–0.9.

#### Differences in thresholds

The smallest biases occurred with VT. Here, the biases of VO_2_ were approximately 0.2 ml/kg/min with a 95% CI of − 1 to 1 ml/kg/min. CV values were approximately 6% and ICC values were between 0.87 and 0.97. There was also no change in bias and CV depending on the length of the step duration.

At lactate thresholds, the biases of VO_2_ were between − 1.8 and 1.2 ml/kg/min with CV between 6 and 17%. Paradoxically, the highest CV values (13% at the 2-mmol threshold and 17% at the 4-mmol threshold) were found in group 1 comparing P0 to P1, despite both protocols apply the same step duration. The lowest CV values (6–9%) with ICC 0.95–0.97 at all thresholds were found in the comparison of P0 to P3, which have the biggest difference in the step duration.

#### Differences in Slopes

The biases of OUES were small at -3% to -1% in all groups. CV values of OUES were < 10% and the ICC values ranged between 0.91 and 0.96.

The biases of VE/VCO_2_ slope were from − 4% to 7%. CV values were < 10% in group 3 and 4, and > 10% in group 1 and 2.

### Test duration and target range

The mean test duration was 11.0 (1.9) minutes for P0, 12.3 (2.6) minutes for P1, 16.0 (2.7) minutes for P2, 17.1 (3.5) minutes for P3 and 13.8 (1.7) minutes for P4. These differences were statistically significant (P0 vs. P1 *p* < 0.002; P0 vs. P2, P3 and P4 *p* < 0.001, respectively). The mean time saved was 4 min per examination for P0. While 70% of the examinations with P0 were in the target range of 8–12 min, this rate was 43% for P1, 13% for P2 and P4 and 3% for P3. Detailed information concerning the test duration is shown in Tables 5 and 6.

**Table 5:** Duration in minutes (mean, standard deviation (SD), median, minimum and maximum)

Analysis variable: test duration in minutes						
Protocol	N	Mean	SD	Median	Minimum	Maximum
P0	120	11.00	1.91	11.00	6.62	18.47
P1	30	12.27	2.61	12.50	8.50	17.83
P2	30	16.03	2.74	16.12	9.05	21.00
P3	30	17.14	3.45	16.84	12.00	25.00
P4	30	13.82	1.69	13.75	10.42	16.92

**Table 6:** % Duration in target range of 8–12 min

Protocol		Duration		
		Under 8 min	8–12 min	over 12 min
P0	N %	3 2.5%	84 70%	33 27.5%
P1	N %	0 0%	13 43.3%	17 56.7%
P2	N %	0 0%	4 13.3%	26 86.7%
P3	N %	0 0%	1 3.3%	29 96.7%
P4	N %	0 0%	4 13.3%	26 86.7%

## Discussion

The goal of this study was to compare performance data collected with different bicycle spiroergometry protocols in adolescents. Results of our study applying different protocols seem to be comparable. The differences of most parameters examined in this investigation were within the biological range of variation.

### Comparability of maximal and submaximal values

For most of the parameters, we found low biases between P0 and P1-P4 and the 95% CIs were narrow.

The low biases for VO_2_peak with < 1 ml/kg/min (with a 95% CI of − 1.8 to 1.4 ml/kg/min) are consistent with other studies showing that the VO_2_peak is independent of exercise protocols in children (Armstrong and McManus 2017; Figueroa-Colon et al. 2000; Sheehan et al. 1987; Welsman et al. 2005), healthy adults (American Thoracic and American College of Chest 2003), heart failure patients (Bensimhon et al. 2008; Corra et al. 2006), and wheelchair users (Leicht et al. 2013).

Information about the comparability of values in submaximal ranges such as lactate or ventilatory thresholds and slopes is lacking in the literature.

Baba et al. have examined the differences between two treadmill protocols in children and adolescents aged 8–18 years (Baba et al. 1999b). With OUES bounds of − 18% to 17% their findings were in line with our data. In a further study, the authors stated that OUES is independent of duration, intensity of exercise tests and motivation (Baba et al. 1999a). Despite these similarities, the limits of agreement to VT were lower in our study (− 20 to 19%) compared to Baba and coworkers (− 31 to 31%).

Kullmer examined adult athletes with different protocols with step durations of 2 and 3 min on the bicycle ergometer. Similar to our data, VO_2_ at IAT were comparable in the protocols (Kullmer 1987).

These findings were also verified by Carta et al. investigating the differences between 1- and 3-min step protocols in adults. They also found no significant difference of VO_2_ observed at anaerobic threshold (Carta et al. 1991).

### Variability of maximal and submaximal values

CV and ICC values in our study largely corresponded to well-defined analytical goals (CV < 10% and ICC > 0.9). Such criteria are commonly used in sport and exercise science (Atkinson and Nevill 1998).

Our results obtained with different protocols are consistent with the values for biological variability on reliability and reproducibility tests performed with repeated testing using the same protocols (American Thoracic and American College of Chest 2003; Armstrong and McManus 2017; Baba et al. 1999a; Johnston et al. 2005; Katch et al. 1982; Keteyian et al. 2010; Tompuri et al. 2016).

The repeatability of VO_2_peak and HRmax was examined by Jonsthon and colleagues in healthy children who performed two exercise tests with the same protocol 3 to 7 days apart (Johnston et al. 2005). Bias, bounds and CV values were in line with our investigation with different protocols.

In a statement on cardiopulmonary exercise testing by the American Thoracic Society (ATS) and American College of Chest Physicians (ACCP), the reproducibility of variables measured during CPET with repeated tests with the same protocol is given with CV values of 4–8% for HRmax, 4–9% of VO_2_peak, 4–14% for O_2_ pulse and 9–13% for VT (American Thoracic and American College of Chest 2003). The CV values in our investigation met these criteria for all these parameters.

CV values of Pmax were between 5.7 and 6.5% in all groups and therefore correspond to the CV value of approximately 6%, which is generally stated for ergometry (Haber 2001).

CV values of the OUES were below 10% corresponding to data published by others (Keteyian et al. 2010; Meyer et al. 1997). CV values of VE/VCO_2_ slope were between 8–14%, and therefore slightly higher than described in the literature with 5% in patients with heart failure (Keteyian et al. 2010).

### Particularities of maximal power and lactate thresholds

As the only one of the investigated parameters, Pmax showed a dependence of size and drift of the bias on the length of the step duration. The drift can be explained by the fact that Pmax is not the physically measured value, but that the wattage in the last step is only fully valid when the step is completed according to its intended duration. If the stage duration is incomplete, the maximum wattage is calculated by linear extrapolation based on time (Haber 2001). This calculation mode therefore leads to a certain degree of inaccuracy in the evaluation of the achieved Pmax in examinations with different step length. In our opinion, the observed differences between tests with a step duration of 1 and 2 or 3 min do not seem to have a significant influence on the fitness assessment in most cases, as the range for normal values in adolescents is relatively wide [14]. Kullmer who examined the difference in Pmax between protocols with 2- and 3-min step durations, also concluded that the difference had no effect on the assessment of individual performance capacity (Kullmer 1987).

In our opinion, caution is warranted when Pmax values are close to the upper or lower limit of the standard value. In these cases, the step duration with which the standard values were created should be considered, otherwise the fitness level could be misjudged. Individual performance follow-up should also only be done with a protocol of the same step duration.

As for the CV values at the lactate thresholds, paradoxically, the best CV at all thresholds were found in group 3 comparing a 1-min (P0) to a 3-min (P3) step duration. The highest CVs were found at the 2- and 4-mmol threshold compared to P1, which is known to be the protocol with the same step duration as P0 (13% at the 2 mmol threshold and 17% at the 4 mmol threshold). These results showed that VO_2_ at lactate thresholds can be stable parameters, even when comparing protocols with different step durations. However, compliance with the preconditions before the test (at least 1 day of sports rest and light meal 2–3 h before the test to ensure full carbohydrate stores) is essential for good comparability (Kullmer 1987).

### Target range and efficiency regarding test duration

We have evaluated the rate of examination reaching the target range of 8–12 min and the temporal efficiency of five examination protocols.

The time target was chosen based on recent recommendations (Buchfuhrer et al. 1983; Hebestreit 2004; Myers et al. 1989; Takken et al. 2017; Whipp et al. 1981; Yoon et al. 2007). The protocols P2, P3 and P4 were largely outside this target range. Although there were statistically significant differences in the duration of examination between these protocols and P0, this had no effect on the comparability of the parameters investigated. This is consistent with current evidence suggesting that CPET should take between 7 and 26 min to establish valid VO_2_peak values (Bishop et al. 1998; Midgley et al. 2008). Studies on the comparability of other CPET parameters with different durations of examination were not found.

Concerning time efficiency, P0 is clearly the favorite with an average time saving of 4 min. The resulting benefit corresponds to approximately 25% less time needed for the examination.

## Conclusion

Comparability between examination protocols with step duration of 1 to 3 min is given for CPET parameters independent of step duration and increments. Protocol-dependent standard values do not appear to be necessary. However, it is recommended that the same protocol should be used for follow-up examinations of the same subject. Only Pmax depends on the step duration, but in most cases, this has no substantial influence on the fitness assessment. When Pmax values are close to the upper or lower limit of the standard value, the step duration with which the standard values were created should be considered, otherwise the fitness level could be misjudged. The extent to which these statements also apply to the comparison with ramp protocols and protocols with a step duration longer than 3 min are subject for further investigations. Concerning time efficiency, P0 is beneficial compared to other protocols.

## Data Availability

The datasets generated and analyzed during the current study are available from the corresponding author on reasonable request.

## Abbreviations

CPET: Cardiopulmonary exercise testing
CV: Coefficient of variation
IAT: Individual anaerobic threshold
ICC: Intra class correlation
HRmax: Maximal heart rate
OUES: Oxygen uptake efficiency slope
P 0–4: Protocol 0–4
Pmax: Maximal power
RER: Respiratory exchange ratio
Rpm: Revolutions per minute
VCO_2_: CO_2_ production
VE: Minute ventilation
VO_2_: Oxygen uptake
VO_2_peak: Peak oxygen uptake
VT: Ventilatory threshold
W/kg: Watt per kilogram body weight
